# Pervasive occurrence of splice-site-creating mutations and their possible involvement in genetic disorders

**DOI:** 10.1038/s41525-022-00294-0

**Published:** 2022-03-18

**Authors:** Narumi Sakaguchi, Mikita Suyama

**Affiliations:** Division of Bioinformatics, Medical Institute of Bioregulation, Kyushu University, Maidashi 3-1-1, Higashi-ku, Fukuoka 812-8582 Japan

**Keywords:** Disease genetics, Genetics research

## Abstract

The search for causative mutations in human genetic disorders has mainly focused on mutations that disrupt coding regions or splice sites. Recently, however, it has been reported that mutations creating splice sites can also cause a range of genetic disorders. In this study, we identified 5656 candidate splice-site-creating mutations (SCMs), of which 3942 are likely to be pathogenic, in 4054 genes responsible for genetic disorders. Reanalysis of exome data obtained from ciliopathy patients led us to identify 38 SCMs as candidate causative mutations. We estimate that, by focusing on SCMs, the increase in diagnosis rate is approximately 5.9–8.5% compared to the number of already known pathogenic variants. This finding suggests that SCMs are mutations worth focusing on in the search for causative mutations of genetic disorders.

## Introduction

Whole-exome sequencing (WES) has been widely used to detect mutations causing genetic disorders in humans. However, WES identifies causative mutations in less than 50% of patients with genetic disorders^1^, and many causative mutations remain unidentified. Causative mutations are detected at low rates because research to date has focused mainly on mutations that disrupt either protein-coding regions or canonical splice sites. The identification of novel causative mutations in genetic disorders will lead to better diagnosis and treatment of patients.

Recently, it has been reported that mutations in intronic or exonic regions can create novel splice sites, resulting in the inclusion of abnormal exons in transcripts^2–5^. This modification often generates premature termination codons (PTCs), giving rise to truncated protein products and often leading to nonsense-mediated mRNA decay (NMD)^6^. Even without the generation of PTCs, the inclusion of abnormal exons may disrupt protein domain structures, producing diseases. However, such splice-site-creating mutations (SCMs) have only been sporadically reported as being involved in genetic disorders to date. Hence, the overall picture of the extent to which SCMs are involved as a cause of genetic disorders is still unclear.

In the present study, to address the above question, we sought to identify SCMs that could be involved in various genetic disorders. We used SpliceAI, a recently devised, highly accurate tool for evaluating the effect of mutations on splice site function^7^. The input data were variants registered in the gnomAD database^8^. We identified 3,942 candidate causative SCMs in 4054 genes listed in the Online Mendelian Inheritance in Man (OMIM) database^9^ (https://www.omim.org/) as being responsible for genetic disorders. We also analyzed existing WES data from ciliopathy patients and identified 38 candidate causative SCMs. We further confirmed that one of the SCMs is exclusively homozygotic in multiple patients. Our results indicate that it is worth considering the possibility that SCMs may be involved in genetic disorders when causative mutations such as disruption of coding regions or splice sites are not found.

## Results

### Identification of splice-site-creating mutations in the causative genes of genetic disorders

**Fig. 1:**
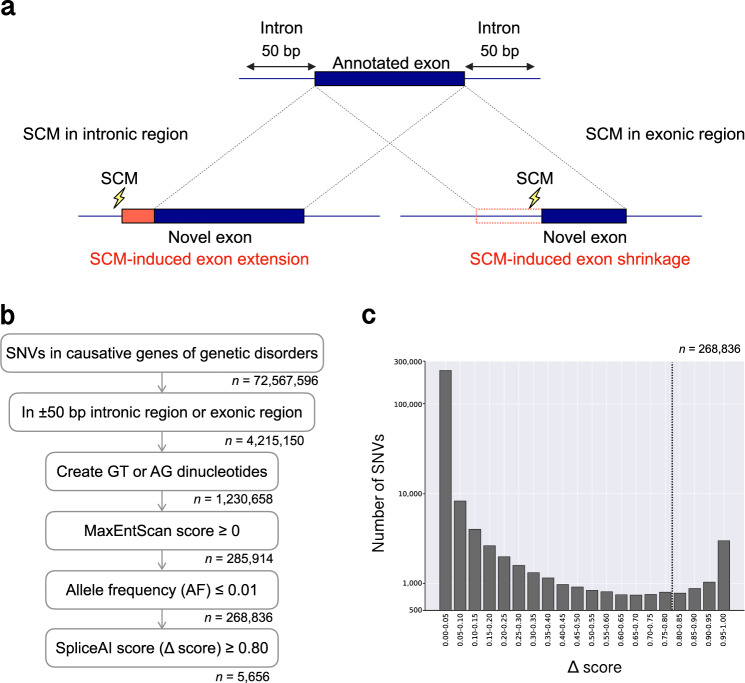
Identification of splice-site-creating mutations in 4054 genes responsible for genetic disorders. **a** Schematic of novel exons by splice-site-creating mutations (SCMs). An exon extension by an SCM in the intronic region (left) and an exon shrinkage by an SCM in the exonic region (right). **b** Workflow for the identification of SCMs. Each number represents the number of SNVs at each step. **c** Distribution of SpliceAI scores (Δscores) for the 268,836 SNVs. The dotted line indicates the cutoff score (0.80) that we adopted for the potential SCMs.

To understand the extent to which splice-site-creating mutations (SCMs) affect the pathogenesis of genetic disorders, we sought to identify those mutations that might shrink or extend an exon in 4054 genes known to be responsible for genetic disorders (Fig. 1a). SCMs were identified using the following steps (Fig. 1b). First, we created a list of genes involved in genetic disorders. From the single-nucleotide variants (SNVs) registered in the gnomAD database (v3.0), we selected only those located in the 4054 genes. The gnomAD database contains more than 800 million SNVs with various allele frequencies and is one of the most comprehensive databases of genetic variation in humans. This step yielded 72,567,596 SNVs. We further selected the SNVs located in intronic regions within 50 bp of an annotated exon–intron boundary or in exonic regions. We introduced this positional cutoff for the intronic SNVs because these genomic intervals are the limits used by conventional WES procedures for identifying mutations^10^. This filtering produced 4,215,150 SNVs. Next, we selected those SNVs that create canonical splice sites, namely, GT or AG, followed by further selection based on the MaxEntScan scores^11^. MaxEntScan is a computational method for evaluating the strength of a splice site. We selected those SNVs that had a MaxEntScan score ≥0, which is a rather relaxed condition. We adopted this cutoff to reduce the number of false negatives. Although the cutoff also increases the number of false positives, such cases can be filtered out in the following steps. We then selected only those SNVs with allele frequencies ≤0.01 in the gnomAD database. We set this condition because we aimed to obtain SNVs that could be causative variants for genetic disorders. After this filtering, the number of SNVs was reduced to 268,836. These SNVs were further evaluated for their potential to be SCMs using the SpliceAI program, which is a highly accurate splice site prediction method. From the distribution of SpliceAI score (Δscore) for the 268,836 SNVs, we found that the recommended cutoff of 0.80 for accurate prediction of splice sites^7^ was located almost at the minimum count of SNVs (Fig. 1c). Based on this observation, we adopted this cutoff and obtained 5656 SNVs that were potential SCMs (Supplementary Table 1).

### Estimation of sensitivity, specificity, and positive predictive value in identifying SCMs

To evaluate the quality of the potential SCMs identified as described above, we compared the potential SCMs to those already identified. To create a set of known SCMs, we used the ClinVar database^12^. We focused on intronic and synonymous exonic SNVs that do not disrupt an annotated splice site and those that create canonical splice sites, because these SNVs are more likely to be SCMs if they are pathogenic. More precisely, we selected those SNVs that (1) were classified as “pathogenic” or “likely pathogenic” in ClinVar, (2) were located in intronic regions within 50 bp of annotated exon–intron boundaries or synonymous sites of exonic regions, and (3) created either GT or AG and did not disrupt the annotated splice sites. There were 775 potential SCMs in ClinVar that met these criteria. Among them, 72 SNVs were within the gene bodies of the 4054 genes known to be responsible for genetic disorders and overlapped with the variants registered in gnomAD. Hence, these 72 SNVs should be known SCMs that our pipeline should detect. Of these 72 SNVs, our pipeline was actually able to identify 49 SNVs as SCMs. This means that the sensitivity of our pipeline is 68.1% (49/72).

To calculate the specificity, first, we artificially introduced the 5656 potential SCMs to the reference genome sequences. Then, RNA-seq data from individuals without these SCMs were mapped onto the artificially mutated reference genome. For this, we used data of ten individuals that have relatively high expression of the genes containing the potential SCMs. This analysis found only three false positives out of the 5656 potential SCMs, indicating the specificity to be 99.9% (5653/5656).

We also measured the positive predictive value (PPV), which is, in this case, the value representing how many of the identified potential SCMs actually form novel splice sites. We used whole blood RNA-seq data of 670 individuals obtained from Genotype-Tissue Expression (GTEx)^13^ (https://gtexportal.org/home/). These are all the individuals with whole blood RNA-seq data. Although mRNAs are thought to be degraded by NMD if alteration of an exon by an SCM generates premature termination codons (PTCs), it has been reported that a substantial number of the transcripts supposed to trigger NMD could be detected in RNA-seq data^14,15^. Among the 5656 potential SCMs, one or more individuals had the mutant allele for 114 SCMs. Of these SCMs, gene expression was confirmed for 53 genes in the RNA-seq data from individuals with the mutant allele. We considered a locus to be expressed if there were at least five junction reads that covered either the novel junction created by the SCM or the annotated junction at that locus. The cutoff was adopted from previous studies^16,17^. Of these 53 loci, 40 had junction reads that supported the novel junctions created by the SCMs. For these 40 loci, we further confirmed that these are true positives using RNA-seq data of all the individuals without the SCMs as the negative control.　Accordingly, we calculated the PPV to be 75.4% (40/53).

### Examples of novel exons induced by SCMs

**Fig. 2:**
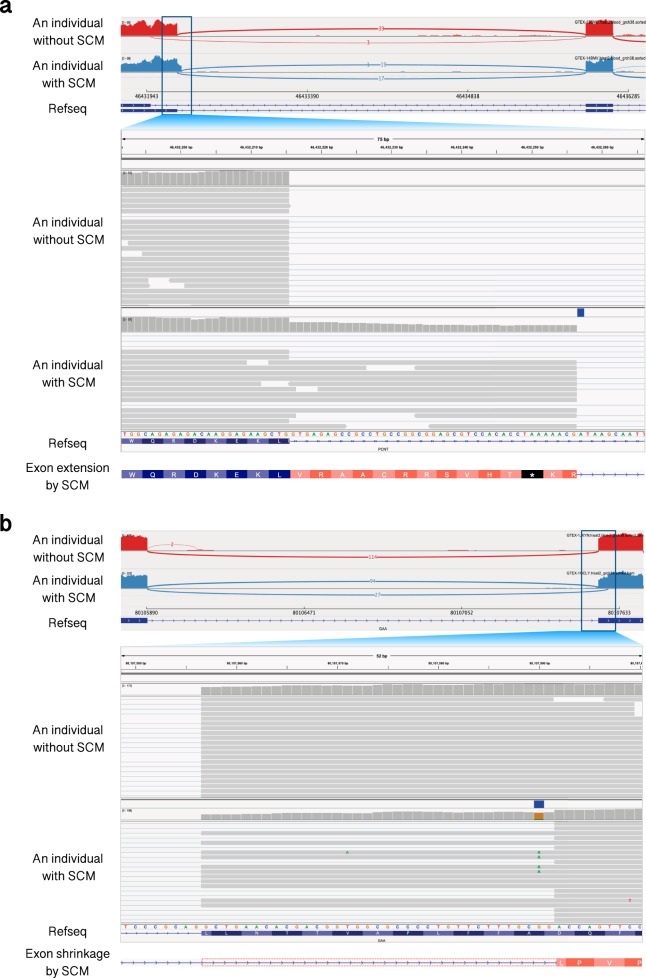
Examples of novel exons induced by SCMs. **a** Exon extension by an SCM in intron 38 of pericentrin (*PCNT*). The upper panel illustrates Sashimi plots^56^ of the extended exon and the downstream exon. The individuals without and with the SCM are shown in red and blue, respectively. Each number represents the number of the junction reads. The lower part of this panel is a close-up view of the genomic region around the SCM. The SCM is indicated at the top of the individual in a blue rectangle. **b** Exon shrinkage by an SCM in exon 5 of acid alpha-glucosidase (*GAA*). The upper panel illustrates Sashimi plots^56^ of the shrunken exon and the upstream exon. The individuals without and with the SCM are shown in red and blue, respectively. Each number represents the number of junction reads. The lower part of this panel is a close-up view of the genomic region around the SCM. The SCM is indicated at the top of the individual in a blue rectangle and indicated in the sequencing reads as mismatched residues.

One of the potential SCMs was found in the intronic region of the pericentrin (*PCNT*) gene, which is known to be a causative gene of the congenital malformation, microcephalic osteodysplastic primordial dwarfism type II (MOPD2)^18^. There was one individual who was heterozygous for this variant in GTEx (GTEX-14BMV). From the RNA-seq data from this individual, we confirmed that the variant was indeed an SCM that induces an exon extension (Fig. 2a). As described in the previous section, such an exon extension was not observed in all the individuals without the SCM. The SCM is an A-to-G transition, which forms the first base of the canonical dinucleotide at the 5′ splice site (5′ss). The novel 5′ss is formed 41 bp downstream of the annotated original 5′ss. Although this locus contains two annotated isoforms, the SCM does not seem to affect the splicing of the shorter isoform because its exon–intron boundary is located 278 bases apart from the SCM. In addition, only a few junction reads exist that support this isoform in both individuals with and without the SCM. According to gnomAD, the allele frequency of this SNV is 1.39505e − 05. The junction allele fraction (JAF) value, which measures the relative usage of the novel splice site based on RNA-seq reads^3^, was calculated to be 0.53. The extended exonic region introduces an in-frame PTC, and hence, it may trigger NMD. To see if the gene expression is reduced as a result of degradation of the transcripts by NMD, we compared the normalized expression levels between the individual with the SCM and 100 randomly selected individuals without the SCM and found that the expression level was not reduced from those without the SCM. Therefore, the SCM, which introduces a PTC in the transcript, might be detrimental due to the production of truncated proteins by the PTC rather than the reduction of the expression by NMD. The SNV has not been reported as a causative factor for MOPD2 so far. Our results suggest that the SNV might be a causative variant for MOPD2, an autosomal recessive monogenic disorder, through exon extension by the SCM, creating a novel 5′ss.

The next example is an SCM found in the exonic region of the acid alpha-glucosidase (*GAA*) gene, which has been reported to be a causative gene of Pompe disease, an autosomal recessive disorder caused by an abnormal accumulation of glycogen in the lysosomes^19^. In GTEx, we also found one individual (GTEX-1QCLY) who was heterozygous for this variant. From the RNA-seq data for this individual, we also confirmed that the variant works as an SCM that induces exon shrinkage (Fig. 2b). As described in the previous section, such an exon shrinkage was not observed in all the individuals without the SCM. The SCM is a G-to-A transition, which forms the first base of the canonical dinucleotide at the 3′ splice site (3′ss). The novel 3′ss is formed 35 bp downstream of the annotated original 3′ss, leading to a frameshift in the downstream exons. The allele frequency reported in gnomAD was 0.00099162. The JAF value was calculated as 0.25. Although the variant was annotated as synonymous by ANNOVAR^20^ and was reported to be “benign/likely benign” or “uncertain significance” in ClinVar, it might be highly damaging because of the frameshift due to the SCM-induced exon shrinkage, which introduces a PTC. In this example, there was also no reduction of the expression in the individual with the SCM, suggesting that the transcript with the PTC will lead to the production of truncated proteins. A pathogenic variant has been reported in ClinVar within the region of the exon shrinkage (Supplementary Fig. 1), and there are also other pathogenic mutations in its downstream exons, further supporting the possible involvement of the SCM in the pathogenesis of Pompe disease.

### Structural characteristics of the identified SCMs

**Fig. 3:**
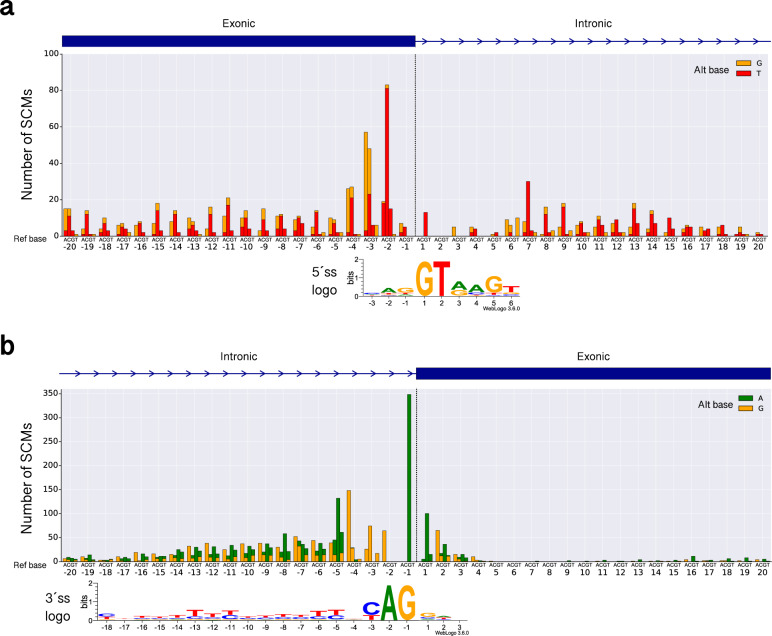
Positional frequency and spectrum of SCMs. **a** 5′ss. **b** 3′ss. The positions of SCMs within 20 bp upstream and downstream of annotated splice sites, respectively, are shown in each panel. The dotted vertical line indicates the annotated original exon–intron boundary. The color codes for alternative bases are shown on the right side of each panel. Note that, in each panel, there are only two alternative bases because we only considered variants that create GT (for 5′ss) or AG (for 3′ss). The SCMs that are more than 20 bp distant from the annotated original exon–intron boundary are not shown. The information content at each splice site is calculated based on the base composition of intron–exon boundaries annotated in GENCODE^48^ (version 29) and converted to the sequence logo representation using WebLogo 3^57^.

We analyzed the structural characteristics of the potential SCMs identified in this study, such as how they were distributed in terms of gene structures. Of the 5656 SCMs, 2218 (39.2%) created 5′ss and 3438 (60.8%) created 3′ss. In intronic regions, 2937 (51.9%) potential SCMs were found, and 2719 (48.1%) were found in exonic regions. For each of the 5′ss and 3′ss, we further analyzed the positional frequencies of the SCMs relative to the annotated splice junctions and the mutational spectrum (Fig. 3). In the 5′ss, SCMs were concentrated in the exonic regions at −3 and −2 (Fig. 3a). The dominant alternate bases were G and T at these sites, respectively, indicating that these substitutions created GYNGYN motifs^21^ often observed in 5′ss. A comparatively smaller number of SCMs were observed in the intronic region next to the annotated exon–intron boundaries, in which the sequence motif for the 5′ss is located. In the rest of the regions, SCMs are fairly uniformly distributed without any positional preferences. In the 3′ss, the most frequent position for SCMs was position −1 in the intronic region, where the second base of the canonical dinucleotide is located (Fig. 3b). Some degree of concentration of SCMs were also observed at positions −5 and −4, with the dominant alternate base being A and G, respectively, creating NAGNAG motifs^22^. In contrast to the 5′ss, SCMs were rarely observed in exonic regions in 3′ss except for the positions closer to exon–intron boundaries. This might be because both a canonical dinucleotide and a polypyrimidine tract are required to function as a novel 3′ss. In the case of SCMs in the intronic regions of 3′ss, an already existing polypyrimidine tract can be utilized to function as a novel 3′ss. This requirement for polypyrimidine tract in 3′ss might explain why the number of SCMs gradually decreased with distance from the original 3′ss.

### Functional consequences of the identified SCMs and their possible contribution to pathogenesis

We systematically analyzed the possible involvement in the pathogenesis of the potential SCMs identified in the 4054 genes known to be responsible for genetic disorders. First, we annotated the identified SCMs in terms of genic regions, such as intronic and nonsynonymous, using ANNOVAR^20^ (Table 1). Then, we further divided them into seven subcategories based on the classification in ClinVar, which reports the associated phenotypes for the variants^12^, to see whether the SNVs identified as SCMs are already classified as pathogenic (Table 1). Only 149 SNVs (2.6%) identified as SCMs are classified as either “pathogenic” (104 SNVs) or “likely pathogenic” (45 SNVs) in ClinVar, and for the rest of the SNVs, most (4986 SNVs) are even not registered in ClinVar and have not yet been reported as pathogenic.

**Table 1:** Classification of the 5656 SCMs using ANNOVAR and ClinVar.

ClinVar	In intronic	Nonsynonymous	Synonymous	Stopgain	ncRNA	5′UTR	3′UTR	Total
Not in ClinVar	2640	1207	709	160	1	222	47	4986
Benign	12	5	18	0	0	0	0	35
Likely benign	26	9	17	0	0	0	1	53
Uncertain significance	142	141	56	1	0	3	1	344
Pathogenic	58	23	5	18	0	0	0	104
Likely pathogenic	31	6	2	6	0	0	0	45
Others	35	27	26	1	0	0	0	89
Total	2944	1418	833	186	1	225	49	5656

**Fig. 4:**
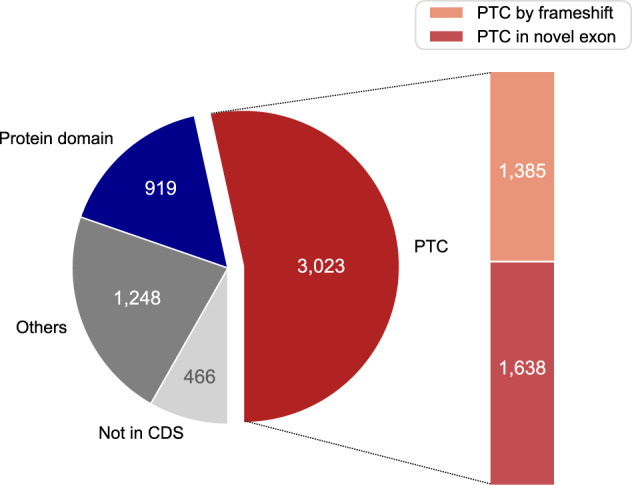
Effect of SCM-induced length alterations on the transcripts and the proteins. The left pie chart shows the functional consequences of the 5656 SCMs identified. “PTC” indicates those that seem to induce NMD by creating PTC. These are further divided into those that create PTCs by frameshifts and those that introduce in-frame PTC in the extended part of the affected exon. “Protein domain” indicates that the coding alteration by the SCM disrupts the protein domain structure. “Not in CDS” indicates that the SCMs are located outside of the protein-coding regions. “Others” indicates SCMs that do not seem to trigger NMD or those that reside outside of the protein domain structures.

As the pathogenicity of most of the SCMs is not clear, to investigate the functional consequences of the mutations, we examined whether the length alteration of exons induced by the SCMs might introduce PTCs that seem to trigger NMD or disrupt protein domain structures (Fig. 4). Among the 5656 exons, whose lengths were altered by the SCMs, 466 had the altered portions outside of the protein-coding regions. In total, there were 3023 SCMs (53.4%) that introduce PTCs either by frameshift (1385 cases) or in the extended exon (1638 cases). Using hmmscan, a tool for assigning annotated protein domains in amino acid sequences^23^, we identified 919 exons (16.2%) in which the altered portions were located within annotated protein domains. Therefore, 3942 SCMs (69.7%) seemed to be associated with disorders, i.e., potentially pathogenic, either by introducing PTC or disrupting protein domains.

We also conducted an analysis of the effect of SCMs on the transcripts using RNA-seq data that were obtained from the SCM containing samples in GTEx. For this, we focused on the 53 loci that we used to calculate PPV in the earlier section. Of these 53 loci, 29 seem to trigger NMD. For each of the 29 loci, we compared the normalized gene expression levels between the individual with the SCM and 100 randomly selected individuals without the SCM to see if the gene expression is reduced as a result of NMD. The result shows that 13 (44.8%) of them have lower expression levels compared to the median values of the individuals without the SCMs, although we could not assess their statistical significance because of the limited number of individuals for each SCM. Even if NMD is not efficiently working on the transcript with a PTC, it will lead to the production of truncated proteins by the PTC, which could have functional consequences.

To further evaluate the contribution of SCMs to the pathogenesis of genetic disorders, we compared the number of potentially pathogenic SCMs with the number of known pathogenic mutations, most of which are identified as nonsense or missense mutations, or those that disrupt existing splice sites. There are 45,505 mutations in ClinVar labeled as “pathogenic” in the 4054 genes known to be responsible for genetic disorders. Among the 3942 potentially pathogenic SCMs, 60 overlapped with pathogenic mutations registered in ClinVar. Although these overlapping mutations are annotated as “pathogenic” in ClinVar because they either are nonsynonymous or disrupt existing splice sites, they might be pathogenic because of their splice-site-creating capability. The rest of the SCMs are not registered in ClinVar, indicating that we can add 3882 more mutations, corresponding to 8.5% (3882/45,505) of the known pathogenic mutations, as potential causes for genetic disorders. Even if we include the “likely pathogenic” mutations in this calculation, the total number is 65,570 together with “pathogenic” mutations, the number of SCMs corresponds to 5.9% of these mutations.

### Identification of SCMs possibly involved in the pathogenesis of ciliopathies from WES data

The above analyses demonstrate the importance of SCMs as causes of genetic disorders. Although WES analysis has widely been applied to identify causative mutations in genetic disorders, it is often difficult to successfully identify the mutations. In some cases, SCMs, which have not actively been considered causes of disorders, can be involved in pathogenesis. Therefore, we searched for SCMs in WES data in which causative mutations are yet to be identified. We focused on ciliopathies because WES data from a large cohort of patients, in which causative mutations have not yet been identified, and their unaffected family members are available in dbGaP^24^ (dbGaP Study Accession: phs000288.v2.p2). Ciliopathies are human disorders in which genes related to primary cilia or motile cilia are mutated. Various phenotypes associated with ciliopathies have been reported throughout the body^25^.

We identified potential SCMs by applying SpliceAI with a cutoff of 0.80 to the variant data obtained from the WES analysis of patients with ciliopathies and their unaffected family members downloaded from dbGaP. We restricted our search space for the SCMs to 434 genes for which involvement in ciliopathies was already suggested^25–39^ (Supplementary Table 2). From these potential SCMs, we selected only those that introduced PTC or disrupted domain structures and then excluded those mutations with AF ≥0.01 or homozygous in normal individuals. We finally obtained 38 potential causative SCMs (Table 2).

**Table 2:** List of 38 potential causative SCMs that may be responsible for ciliopathies.

Nucleotide change	Amino acid change	AF in gnomAD	ss	PTC	Protein domain disruption	Homozygous	Heterozygous	CH variants	Gene	Inheretance	Ciliopathy gene
						No. individuals (Affected)	No. individuals (Affected)				
ENST00000378888.9:c.408 C > T	ENSP00000368166.5:p.Gly136Gly	-	5′ss	No PTC	Disheveled	0 (0)	1 (1)	-	*DVL1*	AD^30^	Established^b^
ENST00000366637.7:c.1269–2 A > G	-	-	3′ss	PTC in novel exon	-	0 (0)	1 (0)	-	*DISC1*	Unknown	Candidate
ENST00000272321.11:c.2079–20 A > G	-	-	3′ss	PTC in novel exon	-	0 (0)	1 (0)	-	*WDPCP*	AR^58^	Established
ENST00000331683.9:c.1527 + 36 A > G	-	0.0007296	5′ss	PTC by frameshift	-	0 (0)	8 (6)	-	*SPAG16*	AD^28,29^	Established^b^
ENST00000295709.7:c.3058–16 C > G	-	-	3′ss	No PTC	HEAT,HEAT_2,HEAT_EZ,Cnd1	0 (0)	1 (1)	-	*STK36*	AR^32^	Established^b^
ENST00000440121.1:c.2197–2 A > G	-	0.000006569	3′ss	PTC in novel exon	-	0 (0)	1 (1)	-	*TTC21A*	Unknown	Candidate
ENST00000301831.8:c.1349–20 T > G	-	0.000486	3′ss	PTC in novel exon	-	0 (0)	3 (1)	-	*ULK4*	Unknown	Candidate
ENST00000420323.6:c.4086 + 27 G > T	-	0.00001314	5′ss	PTC by frameshift	-	0 (0)	1 (1)	-	*DNAH1*	AR^59^	Established
ENST00000420323.6:c.9585 G > T	ENSP00000401514.2:p.Gly3195Gly	-	5′ss	PTC by frameshift	-	0 (0)	1 (1)	-	*DNAH1*	AR^59^	Established
ENST00000330852.9:c.76–9 A > G	-	0.00001977	3′ss	PTC in novel exon	-	0 (0)	2 (1)	-	*RAB28*	AR^60^	Established
ENST00000274192.6:c.654 C > T	ENSP00000274192.5:p.Gly218Gly	0.00007229	5′ss	Do not trigger NMD	Steroid_dh, DUF1295	0 (0)	2 (1)	-	*SRD5A1*	Unknown	Candidate
ENST00000265104.4:c.10593 T > A	ENSP00000265104.4:p.Ser3531Ser	0.00007882	3′ss	No PTC	AAA_9	0 (0)	1 (1)	-	*DNAH5*	AR^61^	Established
ENST00000356031.7:c.3621–3 A > G	-	0.0000657	3′ss	PTC in novel exon	-	0 (0)	2 (2)	-	*SPEF2*	AR^38^	Established^b^
ENST00000356971.3:c.473 A > G	ENSP00000349458.3:p.Asp158Gly	-	5′ss	PTC by frameshift	-	0 (0)	1 (0)	-	*ICK*	AR^62^	Established
ENST00000440747.5:c.1336–8 G > A	-	-	3′ss	PTC by frameshift	-	0 (0)	2 (1)	-	*DNAAF5*	AR^63^	Established
ENST00000404984.5:c.1591–16 T > A	-	-	3′ss	No PTC	IQ	1 (1)	0 (0)	-	*IQCE*	AR^34^	Established^b^
ENST00000409508.7:c.4255–5 A > G	-	0.00001315	3′ss	PTC in novel exon	-	0 (0)	1 (0)	-	*DNAH11*	AR^64^	Established
ENST00000262210.9:c.3206–13 G > A	-	-	3′ss	PTC in novel exon	-	3 (3)	1 (0)	-	*CSPP1*	AR^65,66^	Established
ENST00000242317.8:c.1309 C > T	ENSP00000242317.4:p.Gln437Ter	-	5′ss	PTC by frameshift	-	0 (0)	1 (1)	-	*DNAI1*	AR^67^	Established
ENST00000297814.6:c.2240–7 G > A	-	0.000711	3′ss	PTC in novel exon	-	0 (0)	3 (2)	-	*KIF27*	Unknown	Candidate
ENST00000305242.9:c.820–1 G > A	-	0.0000987	3′ss	PTC in novel exon	-	0 (0)	2 (1)	-	*ARMC4*	AR^68^	Established
ENST00000534099.5:c.1407 G > A	ENSP00000434400.1:p.Pro469Pro	0.00009203	3′ss	No PTC	Tub	0 (0)	2 (1)	-	*TUB*	AR^69^	Established
ENST00000546141.5:c.101–9 G > A	-	0.00001316	3′ss	PTC by frameshift	-	0 (0)	1 (1)	-	*GLI1*	Unknown	Candidate
ENST00000411698.6:c.1504 + 9 C > T	-	-	5′ss	PTC by frameshift	-	0 (0)	1 (1)	-	*MDM1*	Unknown	Candidate
ENST00000553106.5:c.1066–11 G > A	-	0.0003024	3′ss	No PTC	Biopterin_H	0 (0)	4 (2)	-	*PAH*	Unknown	Candidate
ENST00000409039.7:c.10213 A > G	ENSP00000386770.3:p.Ile3405Val	-	5′ss	PTC by frameshift	-	0 (0)	1 (1)	-	*DNAH10*	Unknown	Candidate
ENST00000319980.10:c.1710–4 T > G	-	-	3′ss	No PTC	TPR_8,TPR_1	0 (0)	2 (2)	-	*IFT88*	AR^35^	Established^b^
ENST00000536576.5:c.656 A > G	ENSP00000445067.2:p.Asp219Gly	-	5′ss	PTC by frameshift	-	0 (0)	3 (3)	c.192 G > C^a^ (p.Glu64Asp)	*TTC8*	AR^70^	Established
ENST00000559838.5:c.438 G > A	ENSP00000453449.1:p.Val146Val	-	3′ss	PTC in novel exon	-	0 (0)	1 (1)	-	*MOK*	Unknown	Candidate
ENST00000443035.7:c.1361 T > A	ENSP00000391167.3:p.Leu454Gln	-	3′ss	No PTC	DENN	1 (1)	0 (0)	-	*DENND4A*	Unknown	Candidate
ENST00000379925.7:c.1877A > G	ENSP00000369257.3:p.Asp626Gly	-	5′ss	No PTC	C2-C2_1	0 (0)	1 (1)	-	*RPGRIP1L*	AR^71^	Established
ENST00000338694.6:c.357–1 G > A	-	0.0006964	3′ss	PTC in novel exon	-	0 (0)	2 (2)	-	*TEKT1*	AR^36^	Established^b^
ENST00000570817.5:c.617 C > T	ENSP00000461374.1:p.Ala206Val	0.00003942	5′ss	PTC by frameshift	-	0 (0)	3 (1)	-	*CEP131*	Unknown	Candidate
ENST00000269392.8:c.516–10 G > A	-	-	3′ss	PTC by frameshift	-	0 (0)	2 (2)	-	*CEP131*	Unknown	Candidate
ENST00000315396.7:c.1391 + 7 C > T	-	0.00001974	5′ss	PTC in novel exon	-	0 (0)	2 (1)	-	*CCDC114*	AR^72^	Established
ENST00000278886.10:c.2125 A > G	ENSP00000278886.6:p.Met709Val	0.00001971	5′ss	PTC by frameshift	-	0 (0)	1 (1)	-	*NINL*	AR^73^	Candidate
ENST00000359568.9:c.1872C > G	ENSP00000352572.5:p.Cys624Trp	-	5′ss	PTC by frameshift	-	0 (0)	1 (1)	-	*PCNT*	AR^18^	Candidate
ENST00000357137.8:c.590–16 G > A	-	-	3′ss	PTC in novel exon	-	0 (0)	1 (1)	-	*XPNPEP3*	AR^74^	Established

*AF* allele frequency, *ss* splice site, *CH* compound heterozygous.

^a^All affected individuals have this mutation. This mutation and the SCM seem to be in trans because, for one patient, there is a sibling who has this mutation but not the SCM. The other two patients are siblings in another family.

^b^In a previous study^25^, these genes were labeled as “candidate,” but we changed them to “established” because causative mutations have been identified in these genes in recent reports^28–30,32,34–36,38^.

**Fig. 5:**
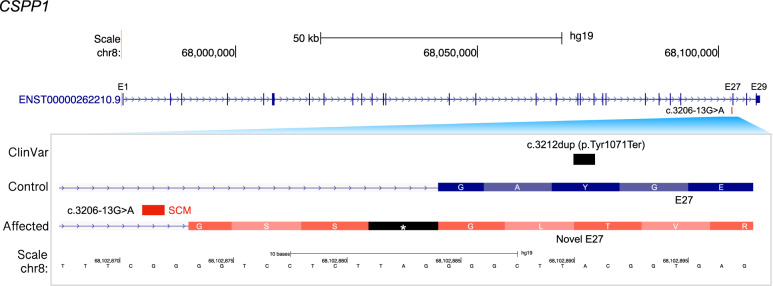
Schematics of exon extension by SCM found in *CSPP1*. The upper panel shows the gene structure of *CSPP1* obtained from the gene annotation data of GENCODE^48^ v24. The lower panel is a close-up view of the SCM and exon 27 (E27). The ClinVar track shows a known pathogenic variant, c.3212dup, for ciliopathy. The gene structure for a control individual is followed by that for an affected individual with the SCM (c.3206–13 G > A) which induces an exon extension. The extended exon contains an in-frame stop codon, shown as an asterisk with a black background.

One of the potential causative SCMs was found in the intronic region of the centrosome and spindle pole associated protein 1 (*CSPP1*) (Fig. 5). The SCM is a G-to-A transition, which forms the first base of the canonical dinucleotide at the 3′ss. The novel 3′ss is formed 11 bp upstream of the annotated original 3′ss (Fig. 5). Due to the SCM, exon 27 (E27) is extended by 11 bp, generating an in-frame stop codon (Fig. 5). This mutation seems to be a rare variant as it is not registered in gnomAD. However, three ciliopathy patients were homozygous for the variant, one unaffected family member was heterozygous, and none of these patients had any known causative mutations, strongly suggesting that the SCM is a causative mutation. Although the SCM was found close to the 3′-end of the gene, exon 27, located just downstream of the SCM, has a known pathogenic nonsense mutation (c.3212dup [p.Tyr1071Ter]) in Joubert syndrome, one of the disorders classified as ciliopathies, providing further evidence that the SCM may be involved in pathogenesis.

**Fig. 6:**
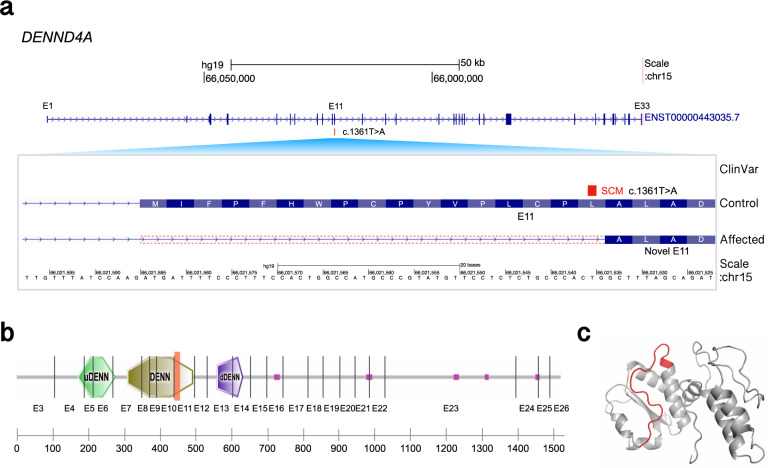
Schematics of exon shrinkage by SCM found in *DENND4A*. **a** The upper panel shows the gene structure of *DENND4A* obtained from gene annotation data of GENCODE^48^ v24. The genomic coordinate is reversed so that the direction of transcription is from left to right. The lower panel is a close-up view of the SCM and exon 11 (E11). The gene structure for a control individual is followed by that for an affected individual with the SCM (c.1361 T > A), which induces exon shrinkage. The dotted box indicates the region of the shrinkage. **b** Effect of the SCM on the protein domain structure. The protein domain architecture is taken from SMART^53^. The red rectangle indicates the 17-amino-acid-long fragment that is coded by the shrunken part of the exon. The vertical line indicates the position of the intron. The pink box indicates the low-complexity region. **c** A three-dimensional protein structural model of the DENN domain constructed by SWISS-MODEL^54^. The 17-amino-acid-long fragment supposed to be deleted by the SCM is shown in red.

Another example of an SCM was found in the exonic region of the DENN domain containing the 4A (*DENND4A*) gene (Fig. 6). The potential causative SCM is a T-to-A transversion, which forms the first base of the canonical dinucleotide at the 3′ss. This mutation is a nonsynonymous substitution that replaces leucine with glutamine and is reported to be “probably damaging” by PolyPhen2^40^. The novel 3′ss is formed 51 bp downstream of the annotated original 3′ss, which results in a 17-amino acid deletion without changing the downstream reading frame (Fig. 6a). Because the deleted segment is located within the DENN domain (Fig. 6b) and spans the three-dimensional protein structure (Fig. 6c), the deletion may affect the domain structure and, hence, its function. The variant is not registered in gnomAD. One ciliopathy patient who does not have any known causative mutations for ciliopathy was homozygous for this variant. No pathogenic mutations have been reported for *DENND4A* in ClinVar. Although the involvement of this gene in ciliogenesis has been predicted by high-throughput genome-wide RNAi screens^39^, direct evidence of its involvement in ciliopathy is yet to be reported. Our finding that the ciliopathy patient has a homozygous potential SCM in this gene provides further evidence supporting its possible involvement in the pathogenesis of ciliopathy.

## Discussion

In this study, to clarify the extent to which SCMs are involved as a cause of genetic disorders, we conducted a systematic search for SCMs in genes known to be responsible for genetic disorders. By applying SpliceAI to variant data from gnomAD and evaluating their ability to form novel splice sites, we were able to comprehensively search for potentially disease-causing SCMs in a variety of genetic diseases without using patient samples. We identified 3942 potentially pathogenic SCMs in the 4054 genes known to be responsible for genetic disorders. Because we adopted relatively simple criteria to assess their functional consequences, some of them may not have functional effects, and hence the number of potentially pathogenic SCMs may have been overestimated. For example, some cases might exist in which the small insertion of peptides by SCMs are located on the surface loop region without any functions of the proteins. Conversely, even among the remaining 1248 SCMs, there can be some damaging mutations caused by other mechanisms than by introducing PTCs or disrupting protein domains, such as those altering disordered regions with a certain function like protein–protein interaction^41,42^. Using the publicly available RNA-seq data obtained from individuals with the potential SCMs, we confirmed that the SCMs function as novel splice sites, as shown in the examples of the *PCNT* and *GAA* loci. By reanalyzing WES data of ciliopathies, with a focus on SCMs, we were able to identify 38 candidate causative mutations.

SCMs might have largely been overlooked because they usually occur in non-conserved regions, where it is difficult to evaluate their functional importance. With the advent of the highly accurate splice site prediction tool SpliceAI, it has become possible to evaluate the mutations occurring in such regions in terms of their potential as splice sites. In addition, by combining large-scale RNA-seq data with corresponding individual genotype data^13^, we were able to confirm the functional impact of some potential SCMs without conducting in vitro molecular biological experiments. However, for most SCMs, their function has not been proven yet because of the lack of RNA-seq data with those mutations. Nevertheless, we believe that our data could be a valuable resource in identifying causative mutations of genetic disorders even in such a situation. For example, suppose a mutation corresponds to one of the potential SCMs, but no other obvious damaging mutations are identified in a disease sample. In that case, the potential SCM is a plausible candidate for a causative mutation. As the possible involvement of such mutations in abnormal splicing can be confirmed by analyzing the structure of transcripts, performing RNA-seq in combination with genotyping can help improve diagnostic rate^1,43–45^.

The number of SCMs associated with the genetic disorders would be a lower limit for two main reasons. The first reason is that we focused only on single-nucleotide substitutions that create either GT or AG dinucleotides in the canonical splice sites. There must be other mutations that strengthen existing cryptic splice sites^3–5^ or create novel splicing regulatory elements, such as exonic splicing enhancers, exonic splicing silencers, intronic splicing enhancers, and intronic splicing silencers^2^. We also excluded mutations that occur in deep intronic regions more than 50 bp from an annotated exon–intron boundary from our analysis. We did not take these mutations into account because either the computational methods currently available to assess potential splice sites^7,11^ are not designed to handle these sites or the accuracy of the predictions is known to be rather low. For example, if we apply SpliceAI to 111 deep intronic SCMs that we identified in our previous study^5^, only 10 had SpliceAI scores ≥0.80, indicating that SpliceAI is not so suitable in detecting deep intronic SCMs. We adopted this rather stringent cutoff in the present study to identify potential SCMs to exclude as many false positives as possible. However, this can be a bias in our list of potential SCMs. The second reason is that, in the search for SCMs in the genes involved in genetic disorders, we focused only on the variants registered in the gnomAD database. As we show in the reanalysis of the exome data for ciliopathies, in which we used all of the variants identified for each sample, 52.6% (20/38) of the potential SCMs are not registered in gnomAD (Table 2). This suggests that the list of 5656 potential SCMs has another bias relying on the gnomAD database. There could be approximately the same number of potential SCMs that are not registered in the current version of gnomAD.

The SCMs identified in this study can be applied in several ways. One such application is the diagnosis of genetic disorders. Diagnoses of genetic disorders may often be delayed because of their rarity in populations and of sometimes weak or no symptoms in early life. If, however, patients with genetic disorders, especially some metabolic disorders, are correctly diagnosed and properly treated in their early life, symptoms may be diminished or almost completely suppressed, leading to improved quality of life in the future^46^. For this purpose, neonatal screening has been developed and is being applied clinically. Although the actual involvement of the potentially pathogenic SCMs has yet to be experimentally proven, adding those SCMs that are confirmed to be pathogenic ones as part of neonatal screening will directly contribute to the improvement of the diagnostic rate of genetic disorders. Another application of the identified SCMs might be as therapeutic targets. Unlike mutations in coding regions and those that disrupt existing splice sites, SCMs can be direct therapeutic targets and treated, for example, by applying antisense oligonucleotides^47^. In the case of disorders caused by SCMs, the underlying pathogenic mechanism is the formation of novel splice sites by mutation. Thus, a well-designed antisense oligonucleotide that could bind and suppress the activity of the novel splice site created by the SCM could be used as a therapeutic agent.

In conclusion, we showed that SCMs might be a rather common type of mutation causing genetic disorders as the number of SCMs is approximately 5.9–8.5% of all currently known pathogenic mutations. We demonstrated that, by focusing on SCMs, reanalysis of existing WES data for which the causative mutations have not yet been identified, can lead to the successful identification of candidate novel causative mutations, suggesting that SCMs should be considered a cause of genetic disorders.

## Methods

### Genomic variants

We downloaded the genomic variant data for 71,702 individuals from the Genome Aggregation Database (gnomAD release 3.0)^8^ in variant call format (VCF). We used 564 million rare SNVs (allele frequency ≤0.01) to identify splice-site-creating mutations (SCMs) caused by genetic disorders. No ethical approval is required as the dataset is allowed to be publicly available.

### Creation of a list of causative genes for genetic disorders

We downloaded the Online Mendelian Inheritance in Man (OMIM) (https://www.omim.org/) data^9^, including names of genes causing human genetic disorders. We used gene structures registered in GENCODE^48^ (version 29) in GTF format. From the GTF file, we extracted the transcripts corresponding to the 4054 causative genes for the genetic disorders. If there were two or more transcript isoforms for a gene, we selected the longest transcript isoform as the representative gene structure.

### Splice site scoring

The strength of the splice site was calculated using the MaxEntScan program^11^. We used the genome sequence segments around the SNV that might form a canonical splice site. For 5′ss scoring, nine bases (six bases in the intronic regions and three bases in the exonic regions of the novel exon–intron boundary created by the candidate SCM) around the SNVs were analyzed using MaxEntScan. For 3′ss scoring, 23 bases (20 bases in the intronic regions and three bases in the exonic regions of the novel exon–intron boundary created by the candidate SCM) around the SNVs were analyzed using the program.

### Sequence-based prediction of SCMs

SpliceAI^7^ was used to detect SCMs. Each SNV in the exonic regions or in introns within 50 bp of an exon–intron boundary was evaluated. We used the SNVs in VCF format, reference genome data (GRCh38.p12), and the gene annotation data from GENCODE (version 29) (https://www.gencodegenes.org/human/release_29.html). SNVs with SpliceAI scores (Δscore) ≥0.80 were considered SCMs.

### Validation using RNA-seq data

To validate whether a candidate SCM indeed functioned as a novel splice site, we used RNA-seq data obtained from individuals who carried the candidate SCM. For this validation, we downloaded variant data of 670 individuals in VCF format and RNA-seq data in BAM format from the GTEx project^13^. These are all the individuals with whole blood RNA-seq data. The datasets are registered in dbGaP as controlled-access data and the Data Access Committee of the National Human Genome Research Institute approved the use of the data for general research use. We have analyzed the data by complying with all relevant regulations. The use of the data does not require the approval of the institutional ethics committee.

To map junction reads precisely to the novel splice site, we created a personal reference genome dataset for RNA-seq data mapping. This step is required because conventional RNA-seq data mapping tools take canonical splice site sequences into consideration, which do not exist in the reference genome. The junction reads originating from SCMs would fail to be correctly aligned. The details of the procedure have already been reported in our previous study^5^. In brief, we created a personal reference genome dataset by applying BCFtools (version 1.9)^49^ to the variant data of the individual and the reference genome data (GRCh38.p12). The RNA-seq data in BAM format for the corresponding individual were converted to FASTQ format using Picard tools (http://broadinstitute.github.io/picard/). Then, the HISAT2 program^50^ (version 2.1.0) was used to map the RNA-seq reads onto the personal reference genome sequence using the default parameters.

### Calculation of junction allele fraction

Junction allele fraction (JAF), a measure of the relative usage of the novel exon–intron junction created by an SCM compared to the annotated junction, was calculated using the following equation^3^:

1 $${{{\mathrm{JAF}}}} = J_n/(J_n + J_a)$$

where *J*_n_ is the number of junction reads supporting the novel splice site and *J*_a_ is the number of junction reads supporting the annotated splice site. JAF values range from 0 to 1, and the closer the value is to 1, the higher is the usage of the novel junction.

### Identification of SCMs in WES data of ciliopathy patients

To analyze ciliopathies, we obtained whole-exome sequencing (WES) data of 2569 patients from dbGaP^24^ (dbGaP Study Accession: phs000288.v2.p2). These data were obtained from inbred families in which the parents were first- or second-degree cousins and there were two or more affected family members. The data included 1773 affected individuals and 796 individuals who were not affected but had two or more affected individuals in their family members. Of the variants obtained from these patients, we used 1,401,863 SNVs located on autosomes and the X chromosome in this study. The datasets are registered in dbGaP as controlled-access data and the Data Access Committee of the National Human Genome Research Institute approved the use of the data for general research use. We have analyzed the data by complying with all relevant regulations. The use of the data does not require the approval of the institutional ethics committee.

A list of 434 ciliopathy-related genes^25–39^ was generated to identify causative SCMs in these genes (Supplementary Table 2). According to a previous study^25^, these genes are classified into two groups: the genes labeled as “established” are those for which the causative mutations have been identified, and the genes labeled as “candidate” are those for which the causative mutations have not been identified but which have been implicated in ciliopathies in other species or at the cellular level. We evaluated all SNVs identified by WES for their potential to be SCMs using SpliceAI with a Δscore cutoff of 0.80. We excluded SCMs with allele frequencies greater than 0.01 or those homozygous in normal individuals in gnomAD. To analyze the ciliopathies, we used GRCh37.p13 as the reference genome and GENCODE (version 24) as the gene annotation data (https://www.gencodegenes.org/human/release_24lift37.html). For each gene, all transcript isoforms were analyzed for possible pathogenicity of the SCMs.

### Data visualization

The Integrative Genomics Viewer^51^ was used to visualize the RNA-seq read mapping and the variant data. The UCSC Genome Browser^52^ was used to visualize the gene annotation data.

### Analysis of protein domain architecture

For the 2167 novel exons that do not seem to be the target of NMD—those that do not disrupt the amino acid sequences either by PTCs or by frameshifts—we examined whether the extension or shrinkage of the sequence by SCMs should affect the protein domains using the hmmscan program^23^. An E-value cutoff of 0.001 was adopted. Protein domain architecture was visualized using the SMART database^53^.

### Modeling a three-dimensional protein structure

To investigate the effect of shrinkage of the exon, which codes a part of the DENN domain of *DENND4A*, on a three-dimensional (3D) protein structure, we constructed a 3D model structure of the DENN domain using SWISS-MODEL^54^. We used the domain sequence obtained from the SMART database as input data. The 3D model structure was visualized using PyMOL^55^ (version 2.1).

### Reporting Summary

Further information on research design is available in the Nature Research Reporting Summary linked to this article.

## Supplementary information

Supplementary Figure 1

Supplementary Table 1

Supplementary Table 2

Reporting Summary

## Data Availability

The genomic variant data for 71,702 individuals are available from Genome Aggregation Database (gnomAD release 3.0)^8^. The GTEx data are available via dbGaP under accession phs000424.v8.p2. The WES data of 2569 ciliopathy patients are available via dbGaP under accession phs000288.v2.p2.

## Code availability

Scripts used in this study are available upon request.
