# Supplementary material for: Pervasive occurrence of splice-site-creating mutations and their possible involvement in genetic disorders
Source: NPJ Genom Med. 2022 Mar 18;7:22. doi: 10.1038/s41525-022-00294-0 (PMC8933504; doi:10.1038/s41525-022-00294-0)
Supplement: Supplementary file 1 — Supplementary Figure 1 [file 41525_2022_294_MOESM1_ESM.pdf]

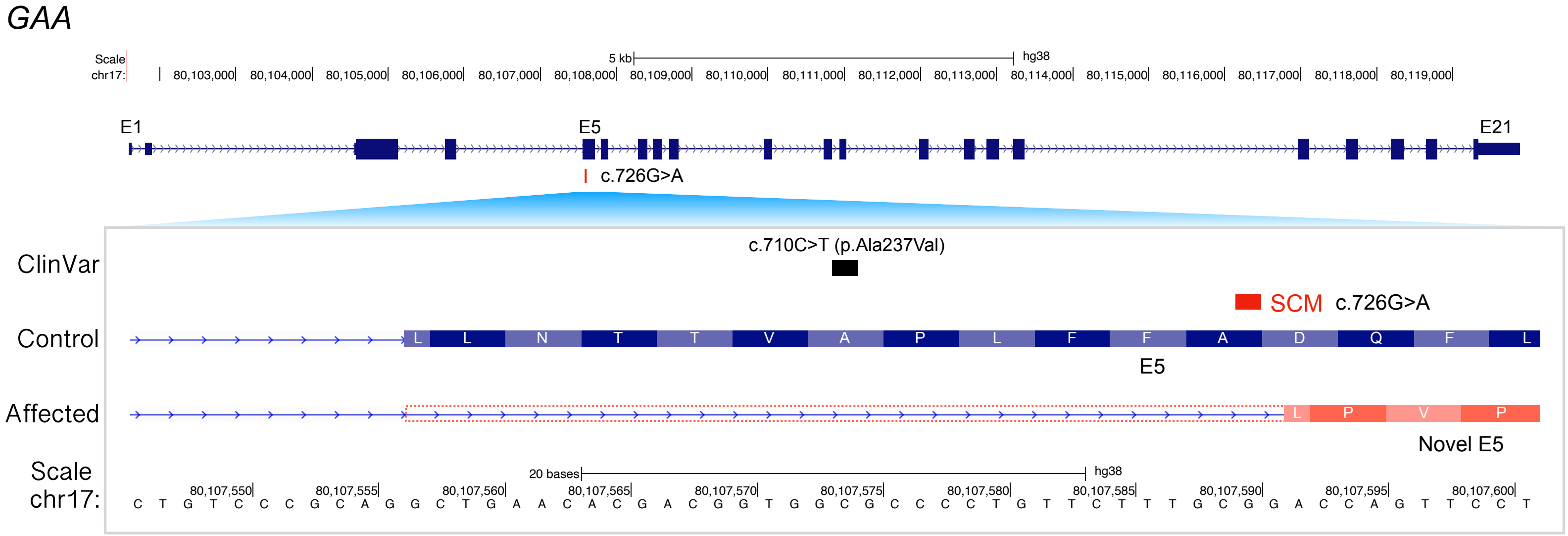

Supplementary Fig. 1 Schematics of exon shrinkage by SCM found in *GAA*.
The upper panel shows the gene structure of *GAA* obtained from the gene annotation data of GENCODE v29. The lower panel is a close-up view of the SCM and exon 5 (E5). The ClinVar track shows a known pathogenic variant, c.710C>T, for Pompe disease. The gene structure for a control individual is followed by that for an affected individual with the SCM (c.726G>A) which induces an exon shrinkage. The dotted box indicates the region of the shrinkage. The shrunken region changes the downstream reading frame.
